# PRGN-2009 and bintrafusp alfa for patients with advanced or metastatic human papillomavirus-associated cancer

**DOI:** 10.1007/s00262-025-04009-z

**Published:** 2025-03-21

**Authors:** Charalampos S. Floudas, Meghali Goswami, Renee N. Donahue, Julius Strauss, Danielle M. Pastor, Jason M. Redman, Isaac Brownell, Evrim B. Turkbey, Seth M. Steinberg, Lisa M. Cordes, Jennifer L. Marté, Maheen H. Khan, Sheri McMahon, Elizabeth Lamping, Michell Manu, Manuk Manukyan, Douglas E. Brough, Amy Lankford, Caroline Jochems, Jeffrey Schlom, James L. Gulley

**Affiliations:** 1 Center for Immuno-Oncology, Center for Cancer Research, National Cancer Institute, National Institutes of Health, Bethesda, MD USA; 2 Dermatology Branch, National Institute of Arthritis and Musculoskeletal and Skin Diseases, National Institutes of Health, Bethesda, MD USA; 3 Laboratory of Pathology, National Cancer Institute, National Institutes of Health, Bethesda, MD USA; 4 Radiology and Imaging Sciences, Clinical Center, National Institutes of Health, Bethesda, MD USA; 5 Biostatistics and Data Management Section, Center for Cancer Research, National Cancer Institute, National Institutes of Health, Bethesda, MD USA; 6 Office of Research Nursing, Center for Cancer Research, National Cancer Institute, National Institutes of Health, Bethesda, MD USA; 7 Clinical Research Directorate, Frederick National Laboratory for Cancer Research, Frederick, MD USA; 8 Precigen Inc., Germantown, MD USA

**Keywords:** Oropharyngeal cancer, Cervical cancer, HPV, Gene therapy, Immune checkpoint blockade, TGF-beta inhibition

## Abstract

**Background:**

This first-in-human phase 1 study (NCT04432597) evaluated the safety and recommended phase 2 dose (RP2D) of PRGN-2009, a gorilla adenoviral-vector targeting oncoproteins E6, E7 (human papillomavirus (HPV)16/18) and E5 (HPV16), as monotherapy (Arm 1A) and combined with the bifunctional TGF-β “trap”/anti-PD-L1 fusion protein bintrafusp alfa (BA; Arm 1B), in patients with recurrent/metastatic HPV-associated cancer.

**Methods:**

Patients with ≥ 1 prior treatment (immunotherapy allowed) received PRGN-2009 (1 × 10^11^ particle units or 5 × 10^11^ particle units, subcutaneously) every 2 weeks for 3 doses, then every 4 weeks (Arm 1A), or PRGN-2009 (RP2D, schedule per Arm 1A) and BA (1200 mg, intravenously) every 2 weeks (Arm 1B). Primary endpoints were safety and RP2D of PRGN-2009; secondary objectives included overall response rate (ORR) and overall survival (OS).

**Results:**

Seventeen patients were treated. In Arm 1A (n = 6) there were no dose limiting toxicities or grade 3/4 treatment-related adverse events (TRAEs), 5 × 10^11^ PU was selected as RP2D, no responses were observed, and median OS (mOS) was 7.4 months (95% CI 2.9–26.8). In Arm 1B (n = 11), grade 3/4 TRAEs occurred in 27% of patients, ORR was 20% for all patients (22% in checkpoint-resistant patients), and mOS was 24.6 months (95% CI 9.6-not reached). Multifunctional HPV-specific T cells were increased or induced de novo in 80% of patients and not impacted by anti-vector antibodies. Higher serum IL-8 at baseline associated with shorter OS.

**Conclusions:**

PRGN-2009 was well tolerated, and immune responses were observed to PRGN-2009. Encouraging anti-tumor activity and OS were noted in the combination with BA arm, consisting mainly of checkpoint-resistant patients.

*Trial Registration* ClinicalTrials.gov Identifier: NCT04432597.

**Supplementary Information:**

The online version contains supplementary material available at 10.1007/s00262-025-04009-z.

## Background

Human papilloma virus (HPV)-associated cancers include cancers of the cervix, vagina, vulva, penis, anus, rectum, and oropharynx, with about 37,000 new HPV-associated cancers diagnosed annually in the U.S [1]. Oropharyngeal cancer (OPC) is the most common HPV-AC in men[1], caused predominantly by HPV16, but also HPV types 35, 33, and 18 [2]. Cervical cancer is the most common HPV-AC in women[1], caused by HPV16 and HPV18 primarily (70%) [3]. The viral oncoproteins E6, E7, and E5 contribute to malignant transformation [4], and constitute immunogenic tumor-specific antigens that may serve as targets for immunotherapies [5].

Immune checkpoint blockade (ICB) disrupts the inhibitory interaction between PD-L1 on tumor cells and PD-1 on T cells and enhances anti-tumor immune responses [6]. Monotherapy with ICBs has shown activity against HPV-associated cancers including OPC [7–9], cervical, [10, 11] and anal [12–14] cancers; however, responses only occur in a minority of patients. Combination of ICB with gene therapy capable of inducing a tumor-specific immune response may improve efficacy.

The PRGN-2009 HPV16/18 targeting gene therapy is based on GC46, a large capacity gorilla adenovirus vector [15] with a low seroprevalence in humans, modified by deletion of viral regions E1 and E4 to abrogate vector replication [16]. The PRGN-2009 molecular design and multi-cytotoxic T lymphocyte (CTL) epitopes grafted onto a protein scaffold were selected for clinical evaluation following evaluation of multiple recombinant gorilla adenovirus constructs for in vitro HPV specific T cell activation and in vivo antitumor efficacy in HPV16 models [17]. The PRGN-2009 antigen design contains 35 non–HLA-restricted CTL epitopes from E6 and E7 (HPV16/18) and E5 (HPV16), including 3 HLA-A2 agonist epitopes of HPV16 that were previously identified and shown to elicit enhanced CTL responses [18], constitutively expressed under control of a cytomegalovirus (CMV) promotor. PRGN-2009 is designed to induce transgene expression in antigen presenting cells to induce a de novo HPV-specific immune response. Preclinical evaluation of PRGN-2009 in mice showed induction of peripheral T cell responses to HPV16/18 peptides, tumor infiltration of CD8 + T cells, and significant reduction in tumor growth rates [17].

Transforming growth factor beta (TGF-β) signaling is upregulated in HPV-AC [19] and induces cancer treatment resistance by suppressing antitumor immune responses [20]. TGF-β signaling inhibition concurrently with ICB may improve outcomes in patients with HPV-associated cancers. Bintrafusp alfa (BA) is a bifunctional TGF-β “trap”/anti-PD-L1 fusion protein composed of the extracellular domain of the TGF-βRII fused to a human IgG1 antibody blocking PD-L1.

In a phase 1 trial of BA in advanced solid tumors (dose escalation and expansion cohorts) conducted by our group BA showed a manageable safety profile [21] and a phase II trial of BA 1200 mg in R/M HPV-associated cancers was conducted. Results from the phase I dose expansion R/M HPV-associated cancers cohort and the phase II were reported jointly, with ORR of 30.5% (95% CI 19.2–43.9) in the immune checkpoint inhibitor-naive population; an additional immune checkpoint inhibitor-resistant population was enrolled in the phase II study, with an ORR of 10% (95% CI 1.2–31.7%) [22]. HPV-specific T cell responses were observed in patients treated with BA monotherapy, particularly in those experiencing objective response or stable disease, suggesting that a combination approach using HPV targeting gene therapy with ICB and TGF-β inhibition to generate HPV-specific T cells may improve clinical activity of BA or potentially other immune checkpoint inhibitors.

We report here the results of a first-in-human double-arm phase 1 clinical trial designed to evaluate the safety and determine the recommended phase 2 dose of PRGN-2009 alone and combined with blockade of PD-L1 and TGF-β with BA in patients with advanced incurable or metastatic HPV-AC.

## Methods

### Study design and participants

This phase 1 nonrandomized clinical trial (NCT04432597) was conducted at the Center for Cancer Research of the National Cancer Institute. The trial was approved by the National Institutes of Health (NIH) Institutional Review Board (IRB) and conducted in accordance with the Declaration of Helsinki [23] and Good Clinical Practice standards. All participants provided written informed consent. Additional follow-up was conducted on long-term follow up and data collection protocols (NIH IRB approved, NCT00451022 and NCT00923065, respectively). Eligible patients were 18 years or older, had histologically confirmed advanced incurable or metastatic HPV-associated cancers, measurable lesions per Response Evaluation Criteria in Solid Tumors (RECIST; version 1.1), Eastern Cooperative Oncology Group (ECOG) Performance Status (PS) of 0, 1, or 2, adequate organ function, and prior ≥ 1 line of systemic treatment; prior immunotherapy was allowed. Tumor HPV testing was not required for enrollment and treatment. HPV status was determined on archival tumor tissue (using MolecularMD RT-PCR, Beckton Dickinson Onclarity HR-HPV PCR) if previous testing results were unavailable (previous testing included also Roche Cobas 4800 PCR, OralDNA Labs PCR).

### Procedures

The trial included two arms, enrolling sequentially: Arm 1A, PRGN-2009 monotherapy dose escalation; Arm 1B, combination PRGN-2009 plus BA. In Arm 1A patients received subcutaneous PRGN-2009 1 × 10^11^ particle units (PU) or 5 × 10^11^ PU, every 2 weeks for three doses, then every 4 weeks. The dose levels were selected based on non-clinical studies by the PRGN-2009 manufacturer and on clinical responses observed in trials of other adenovector based therapeutics, such as the STEP trial [24] and the HVTN trial [25], which also showed that a 1 × 10^11^ PU repeated vaccine regimen was well tolerated. The second dose level (5 × 10^11^ PU) was selected to potentially increase the immunogenicity, based on non-clinical studies by the PRGN-2009 manufacturer. Higher dose levels have not been explored for this adenovector based therapeutic. In Arm 1B patients received subcutaneous PRGN-2009 at the recommended phase 2 dose (RP2D) obtained from Arm 1A, at the same intervals, and intravenous BA, 1200 mg, every 2 weeks. Treatment continued until disease progression, unacceptable toxicity, consent withdrawal, or completion of 1-year treatment (treatment could continue beyond 1 year). Dose reductions were not allowed; interruptions were allowed for the management of adverse events (AEs). There was no intra-patient dose escalation for PRGN-2009 and no crossover between arms. Dose limiting toxicities for the first 3–6 participants in Arms 1A and 1B were defined as specific AEs (listed in Supplemental Methods) possibly attributable to study drugs, occurring within 28 days of starting the PRGN-20090 monotherapy or the combination of PRGN-2009 with BA.

### Outcomes

The primary objectives were determination of safety and the RP2D of PRGN-2009 alone or in combination with BA in patients with recurrent or metastatic HPV-associated cancers. Key secondary endpoints included ORR (defined as the proportion of patients with confirmed complete response (CR) or partial response (PR) by RECIST 1.1) and overall survival (OS, defined as the time from the initiation of study treatment to death from any cause). Exploratory endpoints included the evaluation of HPV16/18 specific T-cell responses in peripheral blood, induction of anti-vector neutralizing antibodies, and circulating immune cell populations, cytokines, and soluble proteins throughout treatment, using methods previously described [26, 27].

### Statistical analysis

Arm 1A used a 3 + 3 dose escalation design with two dose levels. Arm 1B was designed to enroll 10 patients evaluable for response to allow preliminary exploration of the efficacy of the combination, in addition to the safety. Safety was assessed in all patients who received at least one dose of any study drug. Response was assessed in all patients who received at least one dose of any study drug and had disease re-evaluation or exhibited objective disease progression.

Descriptive statistics were used to summarize the study results and Clopper-Pearson 95% confidence intervals (CIs) of ORR were calculated. OS was estimated using the Kaplan–Meier method and differences between survival curves determined by a log-rank test.

Further details can be found in Supplemental Methods.

## Results

### Patient characteristics

Between August 11, 2020, and May 10, 2022, 17 patients with recurrent/metastatic HPV-associated cancers were screened, found eligible, enrolled, and received treatment. Median age for the entire cohort was 61 years (range 43–80 years; 8 (47%) were male. Tumor primary sites included oropharyngeal (7, 41%), cervical (6, 35%), anal (3, 18%), and vaginal (1, 6%). Six patients were enrolled to the PRGN-2009 monotherapy dose escalation arm, and 11 patients were enrolled to the PRGN-2009 plus BA arm. Baseline demographic and disease characteristics are presented in Table 1.

**Table 1:** Participant characteristics

	Arm A	Arm B	Total
	(n = 6)	(n = 11)	(n = 17)
Age, years (median, range)	61 (43–70)	61 (54–80)	61 (43–80)
Female, n (%)	6 (100)	3 (27)	9 (53)
Race, n (%)			
White	2 (33)	9 (82)	11 (65)
Black/African American	3 (50)	2 (18)	5 (29)
Asian	1 (17)	–	1 (6)
Ethnicity, n (%)			
Not Hispanic/Latino	6 (100)	10 (91)	16 (94)
Hispanic/Latinο		1 (9)	1 (6)
Tumor sites, n (%)			
Oropharyngeal	–	7 (63.6)	7 (41)
Cervical	3 (50.0)	3 (27.3)	6 (35)
Anal	2 (33.3)	1 (9.1)	3 (18)
Vaginal	1 (16.7)	–	1 (6)
HPV status, n (%)			
HPV16	3 (50.0)	9 (81.8)	12 (70.6)
HPV18	–	1 (9.1)	1 (5.9)
HPV45		1 (9.1)	1 (5.9)
Negative^b^	2 (33.3)		2 (11.8)
N/A	1 (16.7)	–	1 (5.9)
Previous lines of therapy in metastatic setting, median (range)	2.5 (2–3)	2 (1–4) ^a^	2 (1–4)
ICB exposure, n (%)	6 (100)	10 (90.9)	16 (94)

^a^Two patients with prior HPV16 E7 TCR gene engineered T-cell therapy; one ICB-naïve and one ICB-resistant

^b^Negative for HPV16 or high-risk HPV

All patients had received at least one prior systemic treatment in the recurrent/metastatic setting, and all except one (16, 94%) had received prior ICB. The ICB-naïve patient had a PD-L1-low tumor and had received prior HPV16 E7 T cell receptor (TCR) gene engineered T-cell therapy following standard-of-care treatment with chemotherapy and bevacizumab for cervical cancer. One of the ICB-resistant patients also received prior HPV16 E7 TCR gene engineered T-cell therapy. The median number of prior systemic treatments was 2 (range: 1–4). Fourteen of 17 (82.3%) patients had confirmed HPV + tumors; specifically, 12 (70.6%) had HPV16 + tumors, one (5.9%) had an HPV18 + tumor (oropharynx cancer), and one (5.9%) had an HPV45 + tumor (cervical cancer). Two patients (12%) had tumors that had tested negative for HPV16 or other high-risk HPV genotypes, and one patient (4%) had unknown tumor HPV status (no prior testing and no biospecimen available for testing). Safety was assessed in all 17 patients who received at least one dose of study treatment. Response was evaluated in 16 patients with at least one diagnostic imaging scan beyond baseline (6 in Arm 1A, 10 in Arm 1B).

At the data cutoff date on November 10, 2023, the median potential follow-up duration for all 17 patients was 33.4 months (interquartile range (IQR) 20.8–36.4 months) and no patient was receiving treatment. In Arm 1A, the median duration of treatment was 3.0 months (IQR 1.8–3.7 months; maximum 17.9 months), and the median PRGN-2009 administrations were 5 (range 3–21). In Arm 1B, the median duration of treatment was 3.0 months (IQR 1.8–8.3 months; maximum 23.0 months), and the median PRGN-2009 administrations were 5 (range 1–12).

### Safety

All 17 patients enrolled received at least one dose of treatment. No patient discontinued treatment because of treatment-related adverse events (TRAEs). TRAEs of any grade per arm are listed in Table 2. In the PRGN-2009 monotherapy arm, 5 of 6 patients (83%) had TRAEs, all grade 1 or 2, consisting of injection site reactions, flu-like symptoms, fatigue, and maculopapular rash, symptoms which were mild and tolerable. No dose-limiting toxicities were observed; consequently, dose level 2 was chosen as the RP2D for Arm 1B. The toxicity profile for PRGN-2009 plus BA arm (Arm 1B) included the observed AEs from Arm 1A (PRGN-2009 monotherapy) and those reported from BA monotherapy trials, such as epistaxis, keratoacanthomas, anemia, mouth hemorrhage, upper gastrointestinal hemorrhage [22]. In particular, 11 of 11 patients (100%) had any grade TRAEs, and 3 (27%) reported grade 3 or 4 TRAEs. The most common grade 1 or 2 TRAEs were injection site reactions, flu-like symptoms, fatigue, maculopapular rash, epistaxis, headache, cutaneous keratoacanthomas, fever, decreased lymphocyte count, anemia, and oral hemorrhage. The observed grade 3 or 4 TRAEs were duodenal hemorrhage in 2 (18%) patients and pharyngeal mucositis in 1 patient (9%); all three events were serious AEs, followed by hospitalization. Both patients with duodenal hemorrhage were concurrently receiving non-steroidal anti-inflammatory medications; one patient repeatedly refused blood transfusion for religious convictions and died.

**Table 2:** Treatment-related adverse events

	Arm A (n = 6)		Arm B (n = 11)	
	n (%)		n (%)	
Event	Grade 1–2	Grade 3–4	Grade 1–2	Grade 3–4
Injection site reactions	4 (67)	0	9 (82)	0
Flu-like symptoms	3 (50)	0	6 (55)	0
Fatigue	2 (33)	0	3 (27)	0
Rash, maculopapular	1 (17)	0	2 (18)	0
Epistaxis	0	0	3 (27)	0
Headache	0	0	3 (27)	0
Keratoacanthoma	0	0	3 (27)	0
Fever	0	0	2 (18)	0
Lymphocyte count, decreased	0	0	2 (18)	0
Anemia	0	0	2 (18)	0
Oral hemorrhage	0	0	2 (18)	0
Duodenal Hemorrhage	0	0	0	2 (18)
Pharyngeal mucositis	0	0	0	1 (9)

Reported: grade 1–2 events that occurred in ≥ 10% of patients; all grade 3 and 4 events

### Clinical activity

**Fig. 1:**
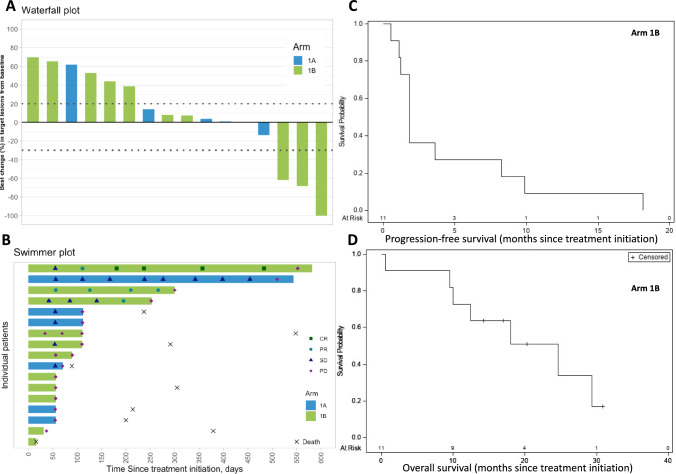
Tumor response and patient survival. **A** Waterfall plot demonstrating best percentage of change in target lesions from baseline among individual patients, across arms. The horizontal dotted lines represent 20% tumor growth and 30% tumor shrinkage. **B** Swimmer plots demonstrating the duration of treatment, for individual patients, across arms (one PR confirmed in Arm 1B). **C** Progression free survival (PFS) in Arm 1B. **D** Overall survival (OS) in Arm 1B; marks on the curve indicate patients who were censored. CR, complete response; PR, partial response, SD, stable disease; PD, progressive disease

In Arm 1A, no objective responses were noted; 4 of 6 patients (66.7%) had stable disease (up to 14.9 months). In Arm 1B, the ORR by RECIST was 20.0% (2/10, 95% CI 2.5–55.6%); median duration of response was 10.1 months. There was a CR in a patient with HPV45 + disease with primary ICB-resistance [28] and a confirmed partial response (PR) in a patient with HPV16 + disease with secondary ICB-resistance [28]; there was 1 unconfirmed PR (uPR) in a patient with HPV16 + disease, ICB-naïve, with prior HPV16 E7 TCR gene engineered T-cell therapy (Table 3). Data on best response, HPV type, PD-L1 status and tumor mutation burden (TMB) are provided for all patients in Supplementary Table 1. Two patients in Arm 1B were treated beyond progression without delayed response. Best change (percentage) in target lesions from baseline and duration of treatment are shown in Fig. 1A, B and longitudinal changes in target lesions from baseline in Supplementary Fig 1. As of November 10, 2023, in Arm 1A, the estimated median OS (mOS) was 7.4 months (95% CI 2.9–26.8 months). In Arm 1B the estimated median progression-free survival (mPFS) was 1.8 months (95% CI 1.1–8.3 months) and the estimated mOS was 24.6 months (95% CI 9.6-not estimable), with a 12-month OS probability of 72.7% (95% CI 37.1–90.3%) and a 24-month OS of 50.9% (95% CI 18.2–76.6%) (Fig. 1C, D).

**Table 3:** Tumor response in evaluable patients

Response^a^	Arm A (n = 6)	Arm B (n = 10)
	n (%)	n (%)
Objective response rate	–	2 (20.0)
95% Confidence interval		(2.5–55.6)
Complete response	–	1 (10.0)^b^
Partial response	–	1 (10.0)^b^
Stable disease	4 (66.7)	2 (20.0)^c^
Progression of disease	2 (33.3)	6 (60.0)^c^

^a^Responses assessed by study radiologist according to the RECIST 1.1. Confirmed responses. An additional unconfirmed PR was observed in Arm B

^b^Immune checkpoint blockade (ICB)-resistant

^c^One patient with prior HPV16 E7 TCR gene engineered T-cell therapy

### HPV-specific peripheral T cell responses

**Fig. 2:**
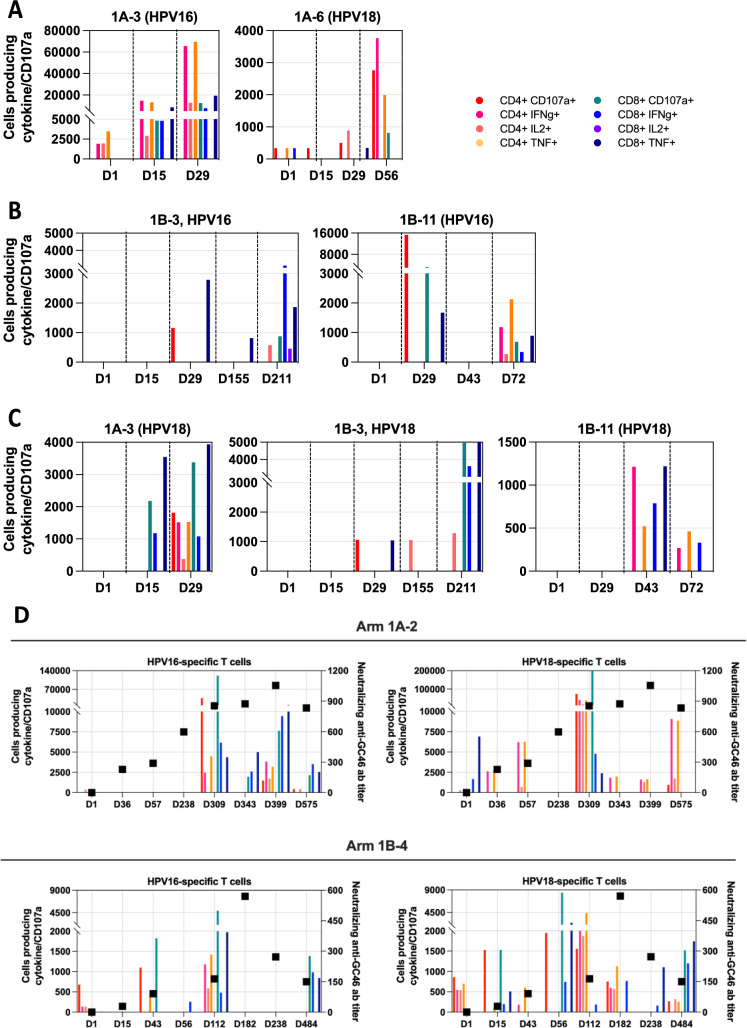
Augmentation and induction of HPV16 and HPV18-specific T cells in patients with or without detectable pre-existing responses. **A** Augmentation of HPV-specific T cell responses in two representative patients where pre-existing responses were detected. Development of HPV-specific T cell responses in patients where no such pre-existing responses were detected for **B** HPV16 and **C** HPV18. The timepoint (in days) evaluated for each patient is indicated along the x-axis, and the CD4 + or CD8 + phenotype readout is indicated in the legend. **D** Development of low levels of neutralizing antibodies does not abrogate development of HPV16 or HPV18 T cell responses. Absolute number of T cells producing cytokines and/or positive for CD107a in response to HPV16 or HPV18 peptide stimulation versus vector-neutralizing antibody titer in a patient from Arm 1A and Arm 1B. CD4 + or CD8 + phenotype readout is indicated in the legend

We evaluated HPV16- and HPV18-specific T cells in patients with available PBMCs before and during treatment. Most patients harbored pre-existing HPV16- and/or HPV18-specific T cells (Supplementary Table 2). All Arm 1A patients (6/6, 100%) developed increased HPV16- and/or HPV18-specific T cells during therapy, with no notable differences in T cell responses observed between the two dose levels of PRGN-2009 (Supplementary Table 2). In Arm 1B, 7/10 (70%) and 7/8 (88%) evaluable patients had increased HPV16- and HPV18-specific T cells, respectively (Supplementary Table 2). Increases in the magnitude and breadth of HPV-specific T cell responses were seen with repeated administration of PRGN-2009 (Fig. 2A).

We also enumerated multifunctional HPV-specific T cells. Five of 6 (83%) patients in Arm 1A and 8/10 (80%) patients in Arm 1B developed threefold or greater increases in multifunctional HPV16- or HPV18-specific T cells during therapy; these responses increased and persisted over time (Supplementary Table 2). Further, in some patients they were absent pre-treatment but detected on-treatment, suggesting the induction of de novo T cell responses (Fig. 2B, C). Further details can be found in Supplementary Results.

### Neutralizing antibody titers

Anti-GC46-vector neutralizing antibodies were either undetectable or detectable at very low titers before PRGN-2009 administration. Neutralizing anti-GC46 antibody titers increased during the initial PRGN-2009 administration every 2 weeks, then stabilized or increase slightly during subsequent monthly administrations (Supplementary Fig 2. Notably, HPV-specific T cells responses were observed concurrently with vector-neutralizing antibodies throughout the PRGN-2009 treatment period (Fig. 2D).

### Expansion of activated CD8 + T cells in the patient experiencing a complete response

**Fig. 3:**
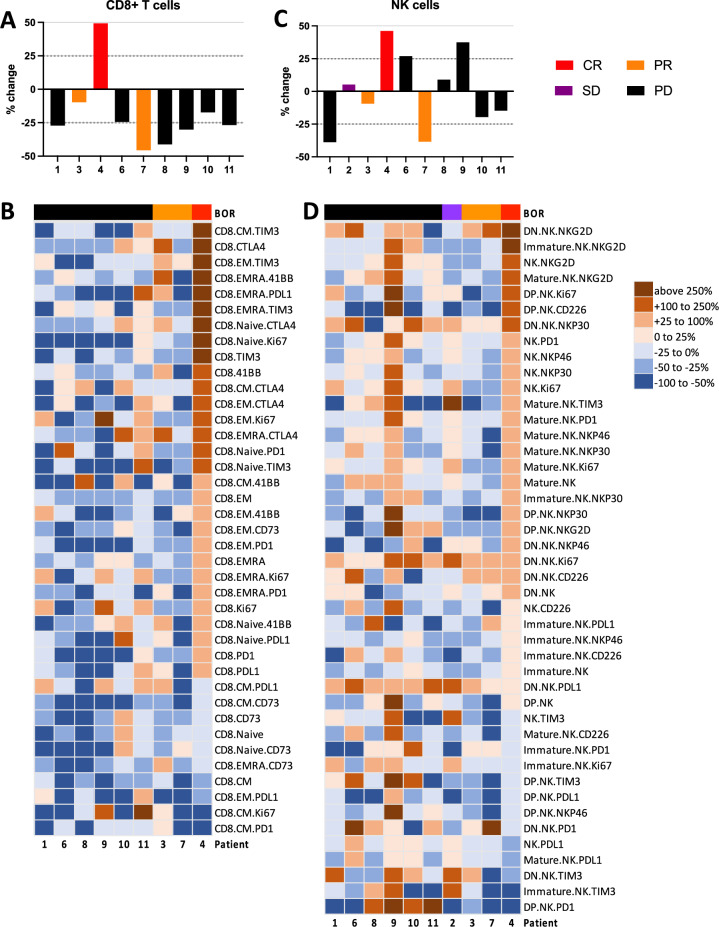
Increase in activated memory T cells and mature NK cells as early as 2 weeks after first PRGN-2009 dose and BA in patient achieving CR. **A** Percent change in total CD8 + T cell frequencies at 2 weeks (D15) compared to baseline. Dotted lines indicate + 25% and − 25% change. **B** Heat map of percent changes at D15 compared to baseline of refined CD8 + T cell subsets based on maturation and activation markers. **C** Percent change in total NK cell frequencies at 2 weeks (D15) compared to baseline. **D** Heat map of percent changes at D15 compared to baseline of refined NK cell subsets based on maturation and activation markers. Binned percent change is indicated in legend. Patients are color-coded by best overall response (BOR) (CR, red; PR, orange; SD, purple; progressive disease (PD), black; including the unconfirmed PR with PR)

We interrogated 158 circulating immune cell populations using high dimensional immunophenotyping at baseline and two weeks after the first dose of PRGN-2009 and BA in the combination arm. Reductions in peripheral frequencies of conventional CD4 + T cells as well as Tregs were seen, accompanied by increases in monocytes and myeloid-derived suppressor cells (MDSCs (Supplementary Fig. 3). Changes in B cells, conventional dendritic cells (DCs), and plasmacytoid DCs were more variable, and there was no association between the direction and/or magnitude of changes in these parental immune cells with response to treatment. Frequencies of peripheral CD8 + T cells decreased compared to baseline in 8/9 (89%) evaluable patients, with one notable exception. The patient who developed a CR during treatment had a 50% increase in circulating CD8 + T cells (Fig. 3A), that were comprised of proliferating CD8 + T cells expressing Ki67, encompassing both naïve and effector T cell states, as well as central and effector subsets positive for the co-stimulatory marker 4-1BB and the activation markers PD-1 and PD-L1 (Fig. 3B). Four patients, including the patient with a CR, had increases in peripheral NK cells (Fig. 3C), that included NK cells expressing proliferative (Ki67) and activation (NKG2D, NKp30, NKp46, PD-1) markers (Fig. 3D). Overall, the patient developing a CR had increases in peripheral CD8 + T cells and NK cells as well as increases in CD4 + T cells, monocytes, MDSCs, cDCs, and pDCs, suggesting a concerted immunological response (Supplementary Fig 4).

### Baseline serum IL-8 levels associated with survival

**Fig. 4:**
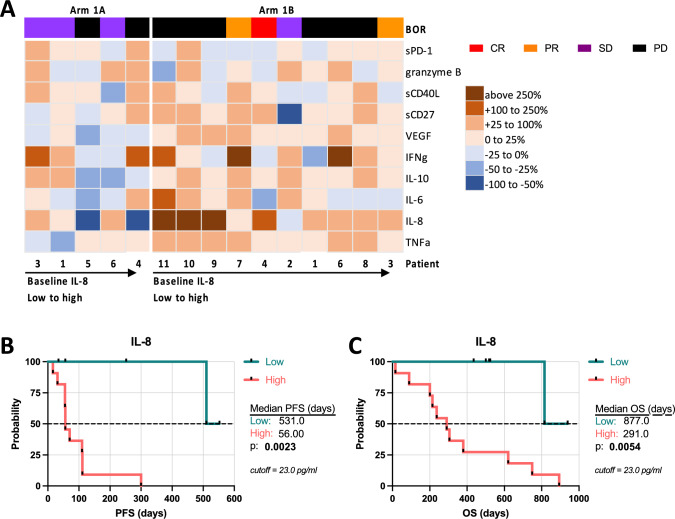
Lower serum concentrations of IL-8 prior to start of treatment correlate with prolonged survival. **A** Heat map of percent changes in serum analytes at day 15 compared to baseline in patients from Arms 1A and 1B. Patients within each arm are ordered from lowest to highest pre-therapy IL-8 serum concentration. Binned percent change is indicated in legend. Patients are color-coded by BOR: CR, red; PR, orange; SD, purple; PD, black; including the unconfirmed PR with PR. Kaplan–Meier survival curves by Mantel-Cox log-rank tests for **B** PFS and **C** OS for all patients treated on study, including Arms 1A and 1B together, stratified by baseline levels of serum IL-8. The cutoff was set at 23.0 pg/ml

We examined circulating levels of cytokines and other circulating factors throughout treatment for changes with therapy and potential associations with clinical outcome. No statistically meaningful changes in measured analytes were seen in the monotherapy arm (1A), while a substantial increase in IL-8 was observed in the combination arm (1B) 2 weeks after treatment with PRGN-2009 and BA (Supplementary Fig 5A). This increase coincided with an increase in absolute neutrophil count at this same timepoint (Supplementary Fig 5B). These increases appeared to be transient, returning to baseline levels beyond this initial timepoint. Fluctuations in many evaluated cytokines and soluble proteins were seen among individual patients (Supplementary Fig 4A); however, neither pre-treatment levels nor percent changes in measured analytes were associated with response to treatment (CR/PR/SD vs. PD). Notably, the three patients with the lowest baseline levels of serum IL-8, who all developed PD with therapy, had steep increases in serum IL-8 of greater than 250% after first treatment (Fig. 4A), suggesting that an early change in IL-8 may reflect disease course.

Associations between pre-therapy concentrations of IL-8 with PFS and OS were also evaluated. A prior study dichotomized patients with solid tumors receiving immune checkpoint inhibitors using baseline levels of IL-8 > 23 pg/ml [29]. Patients who received PRGN-2009 alone or in combination with BA with IL-8 levels below this threshold at baseline had a longer mPFS (531 vs. 56 days, HR: 0.142, 95% CI 0.040–0.499, *p* < 0.01) (Fig. 4B) and mOS (877 vs. 291 days, HR: 0.195, 95% CI 0.062–0.618, *p* < 0.01) than patients above this threshold (Fig. 4C). The treatment arms were also considered separately (Supplementary Fig 5C, D); similar results were seen in the combination treatment arm (Supplementary Fig. 5D). These results highlight the prognostic utility of baseline serum IL-8 concentrations in this setting.

## Discussion

PRGN-2009, a viral vector targeting HPV16/18, alone or in combination with BA, a bifunctional TGF-β “trap”/anti-PD-L1 fusion protein, was well tolerated and showed encouraging activity in patients with HPV-associated cancers. In the monotherapy dose escalation Arm 1A, no dose-limiting toxicities occurred, and PRGN-2009 5 × 10^11^ PU was selected as the RP2D. In the combination treatment Arm 1B, the regimen had a manageable safety profile, with AEs as expected from experience with each agent as monotherapy. In Arm 1A, there was one patient with durable stable disease, but no objective responses were observed. In Arm 1B, the observed ORR was 20.0% (2 of 10 evaluable patients), with one CR (duration of response 8.0 months) and one PR (duration of response 12.2 months), while the mOS was 24.6 months, and the 12- and 24-month OS probability was 72.7% and 50.9%, respectively. In both arms, there was either augmentation of pre-existing or an induction of HPV-specific T cells. Most of these T cell responses were detectable as soon as after three administrations of PRGN-2009, and there was no detectable impact from the development of low levels of anti-vector neutralizing antibodies. The manageable safety profile and immunogenicity of PRGN-2009 alone or in combination with BA and the observed clinical activity of the combination support further exploration of PRGN-2009 in the treatment of HPV-associated cancers alone or as part of combination regimens.

Treatment-related bleeding events occurred in the combination arm of BA with PRGN-2009: any grade in 7 of 11 patients (63.6%), grade < 3 in 5 (45.5%) and grade ≥ 3 in 2 patients (18.2%; both were duodenal hemorrhages, occurring on patients taking NSAIDs). This is comparable to the incidence of treatment-related bleeding events recently reported from a trial of BA monotherapy for patients with recurrent or metastatic cervical cancer, where such events of any grade were noted in 55.5% of patients and of grade ≥ 3 in 17.1% [30].

Monotherapy with ICB in ICB-naïve patients led to ORR 12.2%, mOS 9.4 months [31] and ORR 16.4%, mOS 12.0 months [11] in cervical cancer; ORR 13.8%, mOS 10.1 months in anal cancer [14]; ORR 24%, mOS 8 months in HPV-associated head and neck squamous cell carcinoma (HNSCC) [32]. PD-L1 blockade and TGF-β inhibition with BA in HPV-associated cancers led to ORR 30.5% in ICB-naïve patients, ORR 10%, mOS 3.4 months in ICB-resistant patients, and ORR 21.9% in ICB-naïve patients with cervical cancer [22, 30]. Trials combining HPV therapeutic vaccines with ICB reported ORR 27.6%, mOS 29.2 months in patients with ICB-naïve HPV-associated HNSCC [33] and ORR 11.5% in ICB-resistant OPC [34]; ORR 31.3%, mOS of 16.7 months in ICB-naïve [35, 36] and ORR 21% in patients with cervical cancer [37]; ORR 21%, mOS 17.7 months in ICB-naïve patients with cervical, anal, or penile cancers; [38] ORR 33%, [39] mOS 15.3 months [40] and ORR 22%, mOS 11.0 months [41] in ICB-naïve patients with HPV16 + cancers.

In the present study patients were unselected for PD-L1 expression and all had prior failure of first-line treatment. The trial inclusion criteria did not require prior ICB treatment, but nine of 10 patients included in the dual treatment population were ICB-resistant, with both responding patients having previously failed ICB. Thus, the ORR of 20% reflects a primarily ICB-resistant population. More accurately, the ORR for the ICB-resistant subgroup was 22.2% (2 of 9). Although the sample size is very small and thus results should be interpreted with caution, this ORR is similar to the ORR observed with ICB monotherapy in ICB-naïve patients, and higher than the ORR observed with BA monotherapy for ICB-resistant patients. Notably, two patients with PD in Arm 1B, including the ICB-naïve patient, had received prior TCR-T cell therapy, for which resistance mechanisms include mutation-induced loss or an epigenetically regulated decrease in expression of genes involved in antigen presentation [42]. Tumor cells with such alterations could possibly undergo positive selection during TCR-T treatment, limiting the likelihood of obtaining response with further T cell targeted treatments, such as PRGN-2009 and ICB.

PRGN-2009 is a bivalent vector targeting HPV16/18; of the five HPV vaccines listed above, only MEDI0457 also targets both HPV16/18. In this trial, the CR was observed in an ICB-resistant patient with an HPV45 + tumor. Additionally, in this patient, we observed a robust increase in peripheral blood HPV18-specific T cells, and an early increase in activated CD8 + T and NK cells, indicative of concerted immune activation and cytotoxicity. HPV45 belongs to the high-risk HPV genotypes and is closely related to HPV18, [43] with > 80% sequence homology in the E6 oncoproteins of HPV18 and HPV45 [44]. In addition, sequence homology analysis of epitopes included in PRGN-2009 has shown potential coverage at varying levels for six additional high-risk HPV genotypes, the highest identified for HPV45, with 14 PRGN-2009 epitopes showing cross-coverage with predicted MHC-I epitopes, and three PRGN-2009 epitopes showing cross-coverage with predicted MHC-II epitopes. Therefore, PRGN-2009 may have induced T cell responses against HPV45 and contributed to the favorable clinical outcome in this patient. Importantly, approximately 5% of cervical cancers worldwide are positive for HPV45 [45].

We observed the induction and increase of multifunctional CD4 + and CD8 + HPV-specific T cell responses in peripheral blood following administration of PRGN-2009 either alone (at either dose level) or in combination with BA. Whether this increase translates to enhanced tumor infiltration of HPV-specific T cells is unknown and will need to be assessed in studies with tumor biopsies. Also in this study, we noted unexpected reductions in total CD8 + T cells in most patients early after therapy. Whether this may be due to T‐cell trafficking into and out of the tumor or homeostatic regulation by regulatory cells to suppress an induced immune response will require additional serial testing of PBMC and studies with tumor biopsies. Furthermore, increases in peripheral HPV-specific T cells were accompanied by objective responses in the combination treatment arm but not in the monotherapy arm. This suggests that induction of HPV-specific responses by HPV vaccination may not be sufficient to induce clinically meaningful antitumor activity in the context of advanced, pretreated cancer, and will likely require concurrent administration of other agents such as ICB to address resistance mechanisms. While small numbers, the ORR for ICB-resistant patients with the combination of PRGN-2009 plus BA in this study was higher than that reported with BA monotherapy in ICB-resistant patients, suggesting that the addition of PRGN-2009 may have resulted in improved activity than BA alone.

We also observed an increase in IL-8 levels in serum between baseline and early post treatment timepoints in the combination treatment arm, likely due to BA, considering such an increase was previously reported with BA in patients with HPV-associated cancers [26]. Pre-therapy IL-8 below 23 pg/mL was associated with longer PFS and OS, recapitulating results seen in larger studies of ICB in solid tumors [29, 46].f These increases in serum IL-8 2 weeks post first treatment were particularly marked in a subset of patients developing PD, however neither baseline levels nor changes in IL-8 significantly associated with BOR. The discordance in the relationship between IL-8 and response to treatment versus survival emphasizes the multiple roles of IL-8 in the immune response and warrants further research on the biologic consequences of starting IL-8 levels versus changes in IL-8 with therapy.

Our study is limited by the heterogeneity of the primary HPV-AC site and heterogeneity of HPV status, the small sample size, and the absence of a BA control arm for the dual treatment. Furthermore, research blood samples were limited in the majority of patients, and tumor biopsies were not available, thus limiting the scope of correlative analyses, which are presented in an exploratory context. Additional exploratory studies, including single cell RNA and TCR sequencing, are planned to further elucidate the mode of action of this therapy in the limited number of patients in the current study with additional available research bloods, as well as in ongoing and future clinical trials involving PRGN-2009.

In conclusion, repeat administration of PRGN-2009 is well tolerated and can induce multifunctional HPV-specific T cell immune responses both as monotherapy and in combination with BA. Anti-tumor activity and prolonged patient survival were noted in the combination arm of primarily ICB-resistant patients, suggesting benefit from the combination of PRGN-2009, the PD-L1 ICB, and TGF-β inhibition. Our results also underscore the importance of assessing peripheral blood in immunotherapy trials to describe immunological phenomena that may associate with patient response. A clinical trial exploring the combination of PRGN-2009 with ICB (given BA is no longer currently being developed) in patients with ICB-resistant cervical cancer is underway (NCT06157151).

## Supplementary Information

Below is the link to the electronic supplementary material.

Supplementary file1 (DOCX 22590 KB)

## Data Availability

Deidentified aggregate participant data will be made available at an online repository following publication.

## Abbreviations

95% CI: 95% Confidence interval
AEs: Adverse events
ANC: Absolute neutrophil count
*B2M*: β_2_ Microglobulin
BA: Bintrafusp alfa
CBC: Complete blood cell count
cDCs: Conventional dendritic cells
CMV: Cytomegalovirus
CR: Complete response
CTCAE: Common terminology criteria for adverse events
CTLA-4: Cytotoxic T-lymphocyte associated protein 4
DCs: Dendritic cells
ECOG: Eastern cooperative oncology group
HLA: Human leukocyte antigen
HPV: Human papilloma virus
ICB: Immune checkpoint blockade
IFNγ: Interferon gamma
IgG1: Immunoglobulin G1
IL-2: Interleukin 2
IQR: Interquartile range
MDSCs: Myeloid-derived suppressor cells
MHC: Major histocompatibility complex
mOS: Median OS
mPFS: Median PFS
NCI: National Cancer Institute
NK: Natural killer
OPC: Oropharyngeal cancer
ORR: Overall response rate
OS: Overall survival
PBMCs: Peripheral blood mononuclear cells
PCR: Polymerase chain reaction
PD-1: Programmed cell death protein 1
PD-L1: Programmed death-ligand 1
pDCs: Plasmacytoid dendritic cells
PR: Partial response
PS: Performance status
PU: Particle units
RECIST: Response evaluation criteria in solid tumors
RP2D: Recommended phase 2 dose
SD: Stable disease
TCR: T cell receptor
TGF-β: Transforming growth factor beta
TGF-βRII: TGF-β receptor II
TIM-3: T-cell immunoglobulin and mucin domain 3
TMB: Tumor mutation burden
TNFα: Tumor necrosis factor alpha
TRAEs: Treatment-related adverse events
uPR: Unconfirmed PR
