# Supplementary material for: PRGN-2009 and bintrafusp alfa for patients with advanced or metastatic human papillomavirus-associated cancer
Source: Cancer Immunol Immunother. 2025 Mar 21;74(5):155. doi: 10.1007/s00262-025-04009-z (PMC11928712; doi:10.1007/s00262-025-04009-z)
Supplement: Supplementary file 1 — Supplementary file1 (DOCX 22590 KB) [file 262_2025_4009_MOESM1_ESM.docx]

**Cancer Immunology, Immunotherapy (submitted in 2024) – Charalampos S. Floudas et al.**

**SUPPLEMENTARY FIGURES**


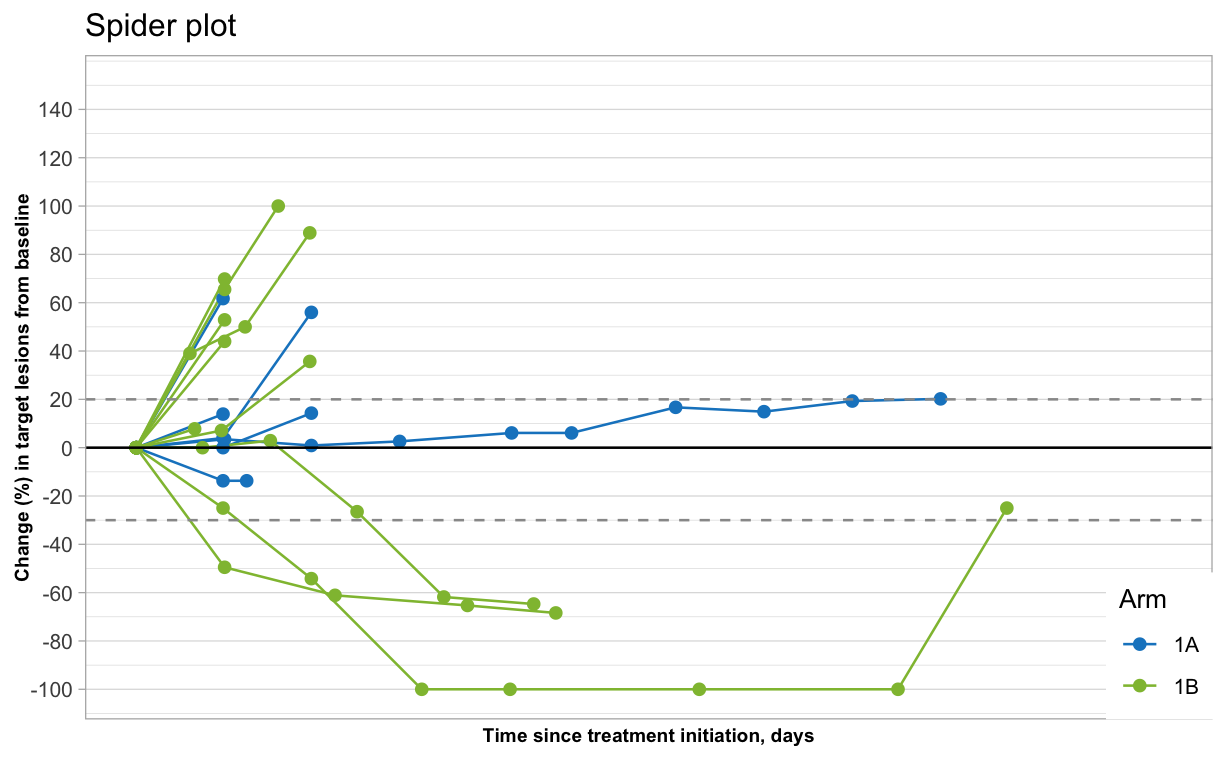


**Supplementary Figure 1** **Spider plot demonstrating the longitudinal percentage change in target lesions from baseline.** The horizontal dotted lines represent 20% tumor growth and 30% tumor shrinkage.


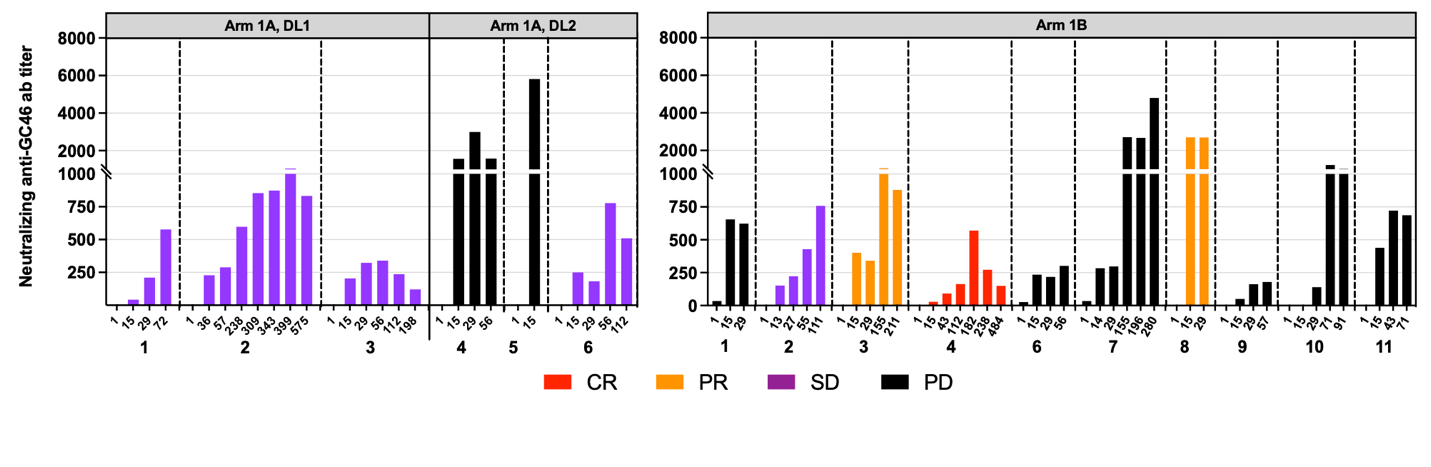


**Supplementary Figure 2 Production of vector-neutralizing antibodies after therapy.** Neutralizing antibody titers against the GC46 adeno-vector before and during treatment from all available timepoints in Arms 1A (left) and 1B (right). Patients are color-coded by best overall response (BOR). (CR, red; PR, orange; SD, purple; PD, black; including the unconfirmed PR with PR).


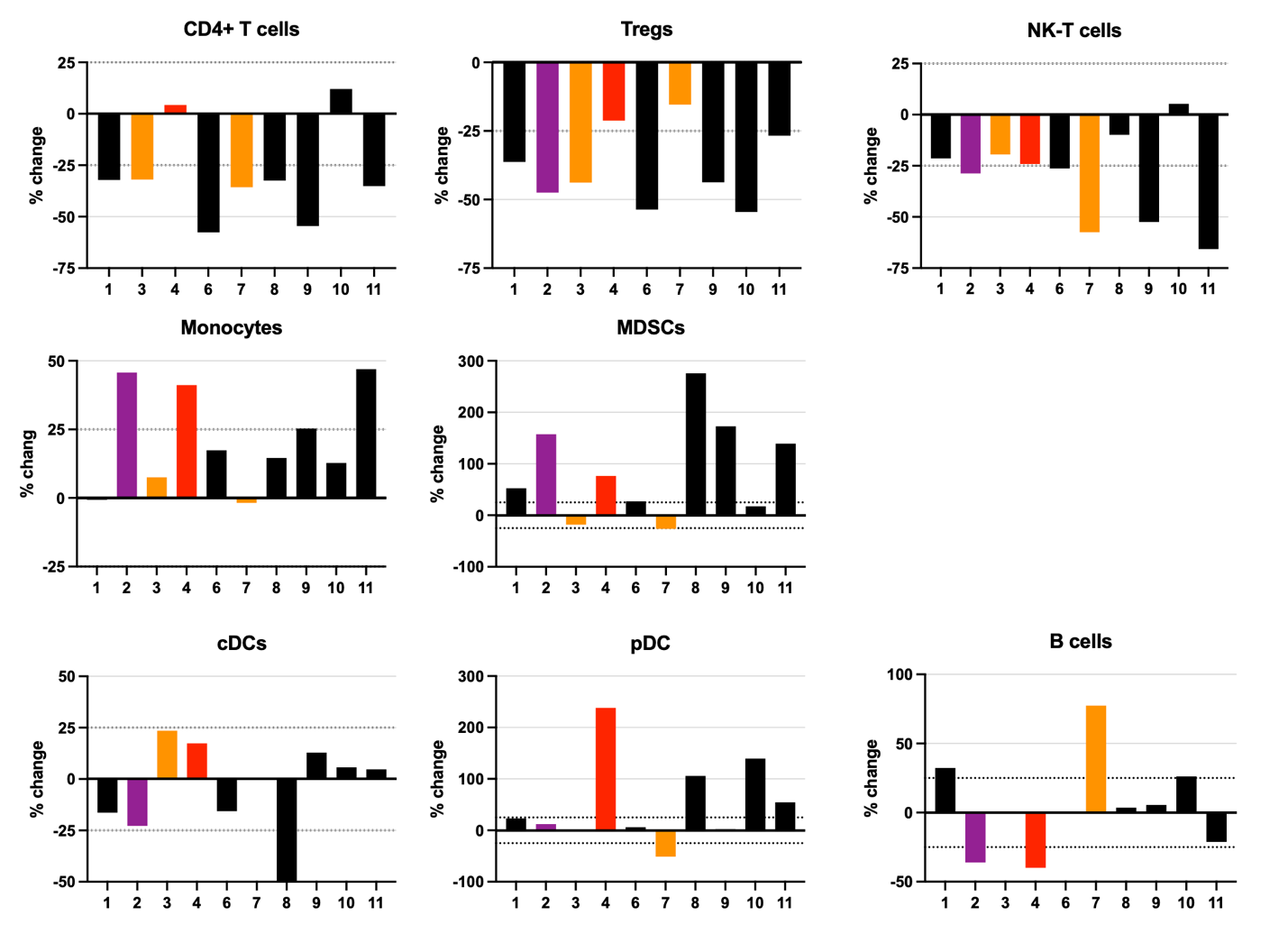


**Supplementary Figure 3. Decreases in circulating conventional CD4+ T cells and Tregs and increases in monocytes and MDSCs 2 weeks after first dose of PRGN-2009 and BA in Arm 1B.** Percent changes in indicated peripheral immune cell subset frequencies at D15 compared to baseline. Patients are color-coded by BOR. (CR, red; PR, orange; SD, purple; PD, black; including the unconfirmed PR with PR). Dotted lines indicate +25% and -25% change.

**
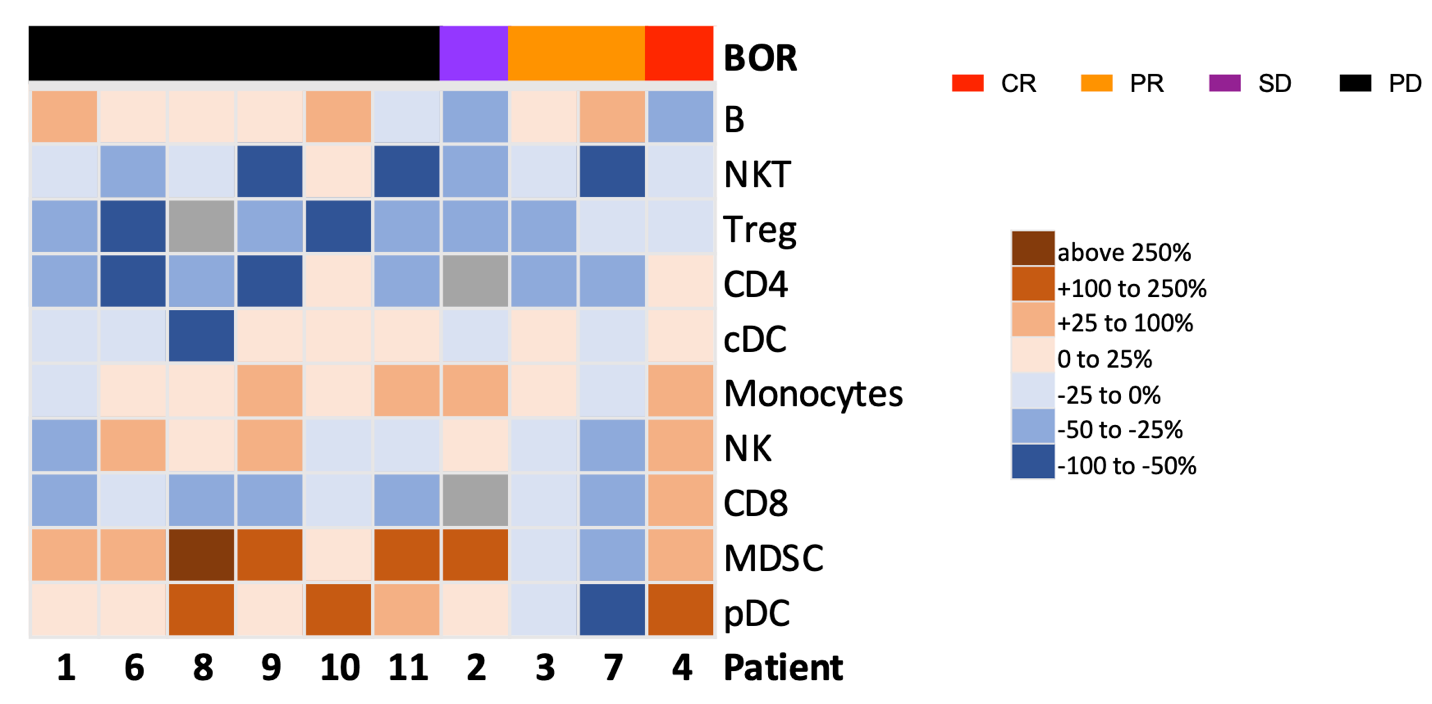
**

**Supplementary Figure 4. Variable changes in parental immune cell subsets 2 weeks after first dose of PRGN-2009 and BA in Arm 1B.** Heat map of percent changes of 10 parental immune cell subsets at D15 compared to baseline in patients. Binned percent change is indicated in legend. Patients are color-coded by BOR. (CR, red; PR, orange; SD, purple; PD, black; including the unconfirmed PR with PR).


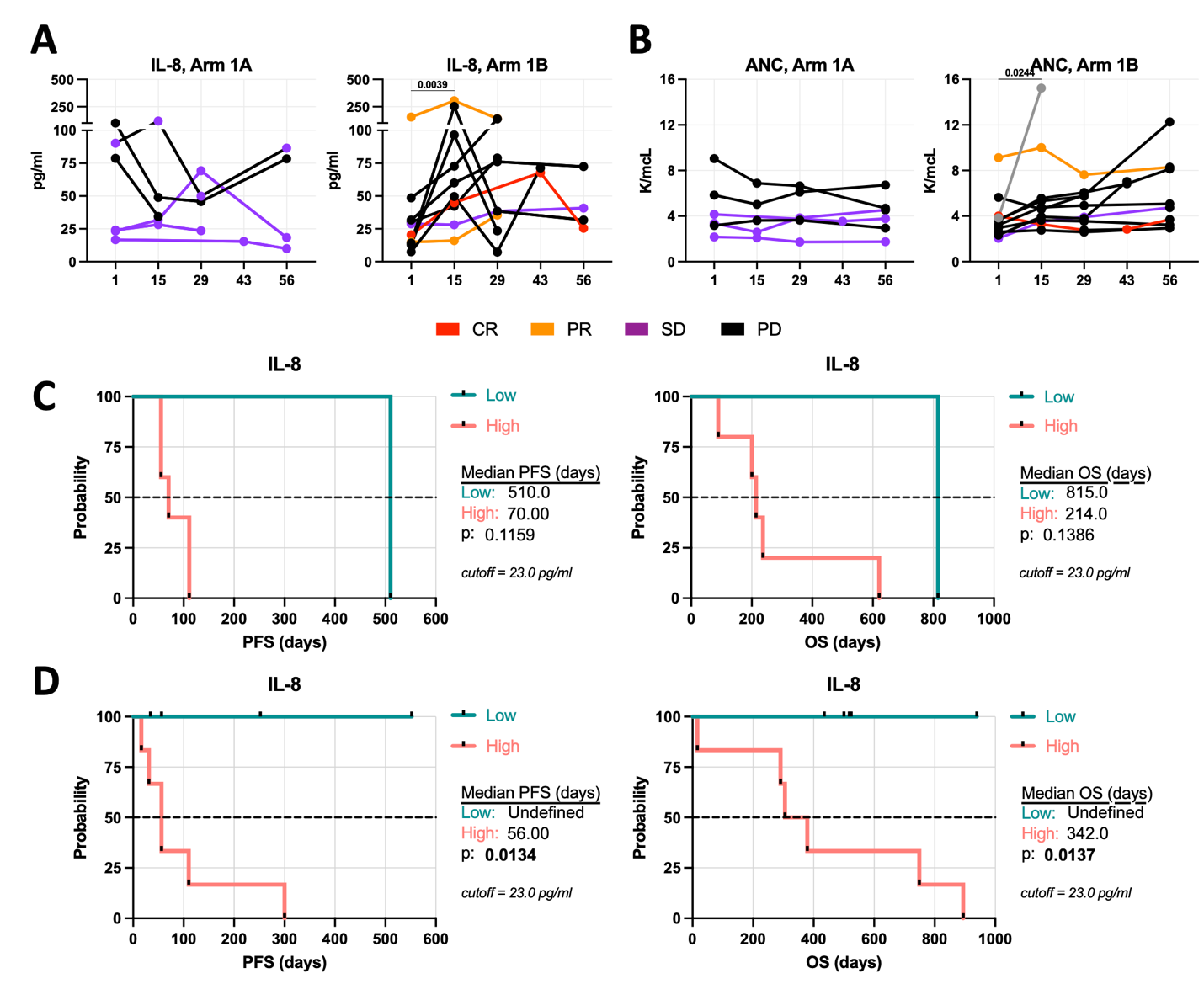


**Supplementary Figure 5. Prognostic value of IL-8 in each arm and changes in serum analytes after one treatment. (a)** IL-8 concentrations and **(b)** ANC through the first two months of treatment in patients treated on Arm 1A and Arm 1B. Patients are color-coded by BOR (CR, red; PR, orange; SD, purple; PD, black; including the unconfirmed PR with PR). Kaplan-Meier survival curves by Mantel-Cox log-rank tests for PFS (left) and OS (right) for patients in **(c)** Arm 1A and **(d)** Arm 1B, stratified by baseline levels of serum IL-8. The cutoff was set at 23.0 pg/ml.

**SUPPLEMENTARY TABLES**

|  | **Primary site** | **Trial arm** | **PRGN-2009 Dose Level** | **BOR** | **HPV**  **type** | **PD-L1** | **TMB** |
| --- | --- | --- | --- | --- | --- | --- | --- |
| 1 | Vaginal | 1A | DL1 | SD | HPV16- | TPS 15% (IHC 22C3) | NA |
| 2 | Cervical | 1A | DL1 | SD | HPV HR- | Low (RNA 1%) | 6 |
| 3 | Anal | 1A | DL1 | SD | HPV16 | 25% (IHC) | 9 |
| 4 | Cervical | 1A | DL2 | PD | HPV16 | Unavailable | NA |
| 5 | Anal | 1A | DL2 | PD | NA | 80% (IHC, SP142) | NA |
| 6 | Cervical | 1A | DL2 | SD | HPV16 | unavailable | 5.5 |
| 1 | OPC | 1B | DL2 | PD | HPV16 | CPS 5% (IHC) | 1 |
| 2 | OPC | 1B | DL2 | SD | HPV16 | TPS 15% (IHC 22C3) | Low |
| 3 | OPC | 1B | DL2 | PR | HPV16 | unavailable | 7.469 |
| 4 | Cervical | 1B | DL2 | CR | HPV45 | unavailable | unavailable |
| 6 | OPC | 1B | DL2 | PD | HPV16 | CPS 1% | 1 |
| 7 | Cervical | 1B | DL2 | SD (uPR) | HPV16 | CPS <1% | 3.2 |
| 8 | OPC | 1B | DL2 | PD | HPV16 | CPS <1% | NA |
| 9 | OPC | 1B | DL2 | PD | HPV16 | TPS <1% | 12.52 |
| 10 | Anal | 1B | DL2 | PD | HPV16 | unavailable | unavailable |
| 11 | OPC | 1B | DL2 | PD | HPV18 | CPS 10% | unavailable |

**Supplementary Table 1.** Patient best response, HPV type, PD-L1 status, TMB.

|  | | | | |
| --- | --- | --- | --- | --- |
| **Measurement** | **HPV antigen** | **Arm 1A** | **Arm 1B DL2** | **All patients combined** |
| **Pre-existing HPV-specific**  **T cell responses** | HPV16 | 6/6 (100%) | 7/10 (70%) | 13/16 (81%) |
|  | HPV18 | 4/6 (67%) | 4/8 (50%) | 8/14 (57%) |
|  | HPV16 or HPV18 | 6/6 (100%) | 8/10 (80%) | 14/16 (88%) |
| **>2-fold increase in HPV-specific T cell response after treatment** | HPV16 | 5/6 (83%) | 7/10 (70%) | 12/16 (75%) |
|  | HPV18 | 5/6 (83%) | 7/8 (88%) | 12/14 (86%) |
|  | HPV16 or HPV18 | 6/6 (100%) | 8/10 (80%) | 14/16 (88%) |
| **>3-fold multifunctional HPV-specific T cell response after treatment** | HPV16 | 4/6 (67%) | 6/10 (60%) | 10/16 (63%) |
|  | HPV18 | 5/6 (83%) | 7/8 (88%) | 12/14 (86%) |
|  | HPV16 or HPV18 | 5/6 (83%) | 8/10 (80%) | 13/16 (81%) |
| **>10-fold multifunctional HPV-specific T cell response after treatment** | HPV16 | 3/6 (50%) | 4/10 (40%) | 7/16 (44%) |
|  | HPV18 | 3/6 50%) | 5/8 (63%) | 8/14 (57%) |
|  | HPV16 or HPV18 | 3/6 (50%) | 5/10 (50%) | 8/16 (50%) |

**Supplementary Table 2** HPV-specific T cell responses.

**SUPPLEMENTARY MATERIAL**

**METHODS**

**Procedures**

History, physical examination, and laboratory testing, including complete blood cell counts (CBCs) and serum laboratory tests, were conducted at baseline and before each treatment. Radiographic evaluation was performed at baseline and every 8 weeks thereafter. AEs were monitored from the initiation of study treatment until 28 days removal from study treatment or until off-study date, whichever came first, and graded using NCI Common Terminology Criteria for Adverse Events, version 5.0. PD-L1 status and tumor mutation burden (TMB) were recorded from prior testing if available.

Dose limiting toxicities for the first 3-6 participants in Arms 1A and 1B were defined as one of the following adverse events possibly attributable to study drugs, occurring within 28 days of starting the PRGN-20090 monotherapy or the combination of PRGN-2009 with BA.

- Any Grade 3 or higher bleeding episode requiring blood transfusion(s).
- Any Grade 4 or higher adverse drug reactions (ADRs) as defined by CTCAE v5.0 and assessed as possibly related to any agent by the Investigator, except for laboratory values that are asymptomatic or resolve to Grade ≤ 1 or baseline grade within 7 days without medical intervention.
- Any Grade 3 ADRs possibly attributed to any agent except for any of the following:
  - Grade 3 flu-like symptoms or fever, as well as associated symptoms of fatigue, headaches, nausea, emesis which can be controlled with conservative medical management.
  - Tumor flare phenomenon defined as local pain, irritation, or rash localized at sites of known or suspected tumor. o Grade 3 Hgb decrease (< 8.0 g/dL) that is clinically manageable with blood transfusions or erythroid growth factor use does not require treatment discontinuation.
  - Grade 3 laboratory values that are asymptomatic or resolve to Grade ≤ 1 or baseline grade within 7 days without medical intervention.
  - Keratoacanthoma and squamous cell carcinoma of the skin. o Any endocrinopathy that can be medically managed with hormone replacement.
  - Any grade 3 adverse drug reaction which can be medically managed with minimal risk to the participants (e.g., placement of a pleural catheter for recurrent inflammatory pleural effusions) and resolves to at least grade one or baseline grade within 72 hours.

**Statistical methods**

Mann-Whitney tests were used to assess statistical differences between 2 groups for a given immune correlate. All statistical tests were two-tailed; reported p-values were not corrected for multiple testing in this hypothesis generating study. Data were analyzed and visualized using R (R Core Team, 2022) with RStudio (v4.2.2), GraphPad Prism (v9.5.1), and SAS v9.4 (Cary, NC).

**Sample collection**

Blood collected in serum separator tubes was centrifuged, and serum was stored at -80°C. To isolate peripheral blood mononuclear cells (PBMCs), blood was collected in sodium heparin tubes and PBMCs isolated by Ficoll-Hypaque density gradient separation. PBMCs were cryopreserved in 90% heat-inactivated human AB serum supplemented with 10% DMSO and stored in liquid nitrogen. CBCs with differential were performed by the NCI Center for Cancer Research.

**HPV-specific T cell analyses**

T cells specific for HPV16/18 were quantified through *in vitro* stimulation assays using peptide pools comprised of overlapping 15-mer peptides encoding E6 and E7 oncoproteins of the HPV16/18 genomes. Peptide pools encoding human leukocyte antigen (HLA) and CEFT (mix of peptides encoding cytomegalovirus, Epstein-Barr virus, influenza, and tetanus toxins) served as negative and positive controls, respectively. All antigen pools were run in parallel for all timepoints collected for a given patient. PBMCs were stimulated for 7 days with peptide with 10ng/ml IL-7 and IL-15 cytokine support on Days 3 and 5. Cells were then rested for 4 days and restimulated overnight with peptide prior to flow cytometric staining. T cell surface expression of CD107a and intracellular expression of IFNγ, TNFα, and IL-2 were used as the determinant of antigen-specificity.

A patient was classified as positive for pre-existing HPV16 or HPV18-specific T cells if they had 250 or more CD4+ or CD8+ T cells positive for CD107a, IFNγ, TNFα, or IL-2 per 1x10^6^ cells as well as a 2-fold or greater increase over the HLA negative control at baseline. Development of an antigen-specific T cell response was defined as 250 or more CD4+ or CD8+ T cells positive for CD107a, IFNγ, TNFα, or IL-2 per 1x10^6^ cells and a >2-fold increase in the number of positive cells at a given timepoint during treatment compared to baseline. Multifunctional HPV16-specific responses were defined as CD4+ or CD8+ T cells expressing 2 or more of the above markers and analyzed in the same manner.

**Neutralizing antibody assays**

Assays for neutralizing antibody were performed as previously described^17,24^. Briefly, serum samples were diluted in Dulbecco’s modified Eagle’s medium at 1:16 to 1:8192, incubated with GC46 vectors expressing the firefly luciferase gene for 1 hour at room temperature, and then used to infect 5x10^4^ A549 cells in triplicate at a multiplicity of infection of 2000 VPU per cell. Twenty-four hours after infection, cells were lysed, and luciferase activity was measured using the ONE-Glo Luciferase Assay (Promega). Samples that resulted in >90% reduction in luciferase activity compared with that in the virus-only control were defined as positive for neutralizing antibodies. The maximum dilution where the serum sample displayed a 90% reduction in luciferase was considered as the endpoint titer.

**Immunophenotyping of PBMCs**

Circulating frequencies of 10 parental immune populations (CD4+ T cells, CD8+ T cells, Tregs, NK cells, NKT cells, cDCs, pDCs, B cells, MDSCs, and monocytes) and 148 further refined immune cell subsets based on markers of maturation and differentiation were evaluated by multicolor flow cytometry using cryopreserved PBMCs from baseline and day 15 (D15) from patients in the combination arm (1B). Samples were acquired with a BD Biosciences LSR Fortessa equipped with five lasers (355-, 407-, 488-, 532-, and 633-nm wavelengths) and DIVA 8 software. Instrument performance was assessed with Cytometer Setup and Tracking beads (BD) as per manufacturer’s recommendation prior to acquisition, and single-color compensation controls were included. Post-acquisition analyses were performed using FlowJo v10 (Tree Star, Inc). After exclusion of doublets, debris, and dead cells, classic immune cell populations and refined subsets were gated and cell frequencies calculated as a percentage of live PBMCs. All gates were established using fluorescence-minus-one (FMO) and single-color controls.

**Serum cytokines and soluble factors**

Serum concentrations of granzyme B and TGFB1 were measured with Quantikine ELISA kits from R&D Systems, and serum VEGF and soluble PD-1 (sPD-1) were assessed using SimpleStep ELISA kits from Abcam. The soluble forms of CD27 (sCD27) and CD40L (sCD40L) were measured using Human Instant ELISA kits from Life Technologies. A multiplexed MesoScale immunoassay platform was used to simultaneously measure serum concentrations of IFNγ, IL-6, IL-8, IL-10, IL-12p70, and TNFα (MesoScale Diagnostics). All assays were performed according to manufacturer’s protocol.

**RESULTS**

**HPV-specific peripheral T cell responses**

In preclinical studies, PRGN-2009 led to increases in both HPV16 E6/E7-specific CD4+ and CD8+ T cell responses.[19] Thus, we evaluated if increases in HPV16 and HPV18-specific T cells occurred in patients after treatment with PRGN-2009, administered either alone or in combination with BA, through *in vitro* stimulation assays of PBMCs using peptide pools encoding HPV16 and HPV18 E6/E7 oncoproteins. Most patients harbored pre-existing T cell reactivity towards HPV16 and/or HPV18 (**Table S2**). In the monotherapy arm, 6/6 (100%) patients developed increased HPV-specific T cells against either HPV16 or HPV18, at any timepoint evaluated during therapy defined as 250 or more cytokine-producing or CD107a+ CD4+ or CD8+ T cells per 1x10^6^ PBMCs, as well as a >2-fold increase in the number of positive T cells compared to baseline. In the combination arm, 7/10 (70%) and 7/8 (88%) evaluable patients mounted increased antigen-specific T cell responses against HPV16 and HPV18, respectively (**Table S2)**.

The percentage of patients developing HPV-specific T cell responses greater than baseline increased with the number of PRGN-2009 administrations. Two weeks after the first PRGN-2009 dose, 6/15 (40%) and 5/13 (38%) evaluable patients had developed T cell response to HPV16 and HPV18, respectively. This increased to 5/8 (63%) against HPV16 and 8/8 (100%) against HPV18 one month after the third dose of PRGN-2009. Additionally, both the magnitude and breadth of HPV-specific T cells were boosted with repeated vaccination in some patients with detectable but low levels of pre-existing HPV-specific T cell responses (**Fig. 2A**). We also enumerated multifunctional T cells, defined as HPV16 or HPV18-specific CD4+ and CD8+ T cells expressing at least 2 of the following markers: CD107a, IFNγ, TNFα, or IL-2. Five of 6 (83%) patients in the monotherapy arm and 8/10 (80%) patients in the combination arm developed 3-fold or greater increases in multifunctional HPV16 or HPV18-specific CD4+ and/or CD8+ T cells during therapy over baseline. Moreover, 50% of patients in both arms 1A and 1B had a 10-fold or greater increase in multifunctional T cells during treatment (**Table S2**). In two patients with available samples beyond one year, HPV-specific T cell responses were still detectable, indicating long-term persistence of responses. In 3 patients without detectable HPV16-specific T cells and 4 patients without detectable HPV18-specific T cells prior to therapy start, there was induction of such responses of varying magnitude and breadth in CD4+ and/or CD8+ T cell compartments during treatment, suggesting the induction of de novo T cell responses against HPV16 and HPV18 targets after administration of PRGN-2009 (**Fig. 2B, C**).

**Trial Participation Eligibility Criteria (initial approval, 2019-08-09)**

Inclusion criteria:

1. Subjects with cytologically or histologically confirmed locally advanced not amenable to potentially curative local therapies or metastatic HPV associated malignancies:
   1. Cervical cancers;
   2. p16+ Oropharyngeal cancers;
   3. Anal cancers;
   4. Vulvar, vaginal, penile, and squamous cell rectal cancers;
   5. Other locally advanced or metastatic solid tumors (e.g., lung, esophagus) that are known HPV+.
2. Subjects must have measurable disease, per RECIST 1.1.
3. Participants must have received one prior line of systemic chemotherapy in the recurrent/metastatic setting as well as checkpoint blockade therapy in tumors with FDA approval (head and neck squamous cell cancer and PDL1+ cervical cancer). Exceptions to this include participants not eligible to receive standard therapy.
4. Men or Women; Age >18 years.
5. ECOG performance status < 2.
6. Adequate hematologic function at screening, as follows:
   1. Absolute neutrophil count (ANC) ≥1 x 109/L;
   2. Hemoglobin ≥ 9 g/dL;
   3. Platelets ≥ 75,000/microliter.
7. Adequate renal and hepatic function at screening, as follows:
   1. Serum creatinine ≤ 1.5 x upper limit of normal (ULN) OR Measured or calculated creatinine clearance ≥ 40 mL/min for participant with creatinine levels > 1.5 X institutional ULN (GFR can also be used in place of creatinine or CrCl);
   2. Bilirubin ≤ 1.5 x ULN OR in subjects with Gilbert’s syndrome, a total bilirubin ≤ 3.0 x ULN;
   3. Alanine aminotransferase (ALT) and aspartate aminotransferase (AST) ≤ 2.5 x ULN, unless liver metastases are present, then values must be ≤ 3 x ULN).
8. The effects of the immunotherapies the developing human fetus are unknown. For this reason and because immunotherapeutic agents as well as other therapeutic agents used in this trial are known to be teratogenic, women of child-bearing potential and men must agree to use highly effective contraception (hormonal or barrier method of birth control; abstinence) prior to study entry and up to 2 months following the last dose of any study treatment. Should a woman become pregnant or suspect she is pregnant while she or her partner is participating in this study, she should inform her treating physician immediately.
9. Participants serologically positive for HIV, Hep B, Hep C are eligible as long as the viral loads are undetectable by quantitative PCR. HIV positive participants must have CD4 count ≥ 200 cells per cubic millimeter at enrollment, be on stable antiretroviral therapy for at least 4 weeks and have no reported opportunistic infections or Castleman’s disease within 12 months prior to enrollment.

**Exclusion Criteria**

1. Patients with prior investigational drug, chemotherapy, immunotherapy or any prior radiotherapy (except for palliative bone directed therapy) within the past 28 days prior to the first drug administration except if the investigator has assessed that all residual treatment-related toxicities have resolved or are minimal and feel the patient is otherwise suitable for enrollment.
2. Major surgery within 28 days prior to the first drug administration (minimally invasive procedures such as diagnostic biopsies are permitted).
3. Known active brain or central nervous system metastasis (less than a month out from definitive radiotherapy or surgery), seizures requiring anticonvulsant treatment (<3 months) or clinically significant cerebrovascular accident (<3 months). In order to be eligible patients must have repeat CNS imaging at least a month after definitive treatment showing stable CNS disease. Patients with evidence of intratumoral or peritumoral hemorrhage on baseline imaging are also excluded unless the hemorrhage is grade ≤ 1 and has been shown to be stable on two consecutive imaging scans.
4. Pregnant women are excluded from this study because these drugs have not been tested in pregnant women and there is potential for teratogenic or abortifacient effects. Because there is an unknown but potential risk for adverse events in nursing infants secondary to treatment of the mother with these immunotherapies, breastfeeding should be discontinued if the mother is treated on this protocol.
5. Active autoimmune disease that might deteriorate when receiving an immunostimulatory agent with exception of:
   1. Diabetes type I, eczema, vitiligo, alopecia, psoriasis, hypo- or hyperthyroid disease or other mild autoimmune disorders not requiring immunosuppressive treatment;
   2. Subjects requiring hormone replacement with corticosteroids are eligible if the steroids are administered only for the purpose of hormonal replacement and at doses ≤ 10 mg of prednisone or equivalent per day;
   3. Administration of steroids for other conditions through a route known to result in a minimal systemic exposure (topical, intranasal, intro-ocular, or inhalation) is acceptable;
   4. Subjects on systemic intravenous or oral corticosteroid therapy with the exception of physiologic doses of corticosteroids (≤ the equivalent of prednisone 10 mg/day) or other immunosuppressives such as azathioprine or cyclosporin A are excluded on the basis of potential immune suppression. For these subjects these excluded treatments must be discontinued at least 1 weeks prior to enrollment for recent short course use (≤ 14 days) or discontinued at least 4 weeks prior to enrollment for long term use (> 14 days). In addition, the use of corticosteroids as premedication for contrast-enhanced studies is allowed prior to enrollment and on study.
6. Subjects with a history of serious intercurrent chronic or acute illness, such as cardiac or pulmonary disease, hepatic disease, bleeding diathesis or recent (within 3 months) clinically significant bleeding events, or other illness considered by the Investigator as high risk for investigational drug treatment.
7. History of second malignancy within 3 years of enrollment except for the following: adequately treated localized skin cancer, cervical carcinoma in situ, superficial bladder cancer, other localized malignancy which has been adequately treated or malignancy which does not require active systemic treatment (e.g. low risk CLL). For patients enrolled on the phase I portion of the protocol a second HPV driven malignancy is allowed.
8. Subjects with a known severe hypersensitivity reaction to monoclonal antibodies (grade >/= 3 NCI-CTCAE v5) will be evaluated by the allergy/immunology team prior to enrollment.
9. Receipt of prior lymphodepleting chemotherapy (e.g. cyclophosphamide, fludarabine) or any organ transplantation requiring ongoing immunosuppression.
